# A role for endothelial nitric oxide synthase in intestinal stem cell proliferation and mesenchymal colorectal cancer

**DOI:** 10.1186/s12915-017-0472-5

**Published:** 2018-01-10

**Authors:** Jon Peñarando, Laura M. López-Sánchez, Rafael Mena, Silvia Guil-Luna, Francisco Conde, Vanessa Hernández, Marta Toledano, Victoria Gudiño, Michela Raponi, Caroline Billard, Carlos Villar, César Díaz, José Gómez-Barbadillo, Juan De la Haba-Rodríguez, Kevin Myant, Enrique Aranda, Antonio Rodríguez-Ariza

**Affiliations:** 1 Instituto Maimónides de Investigación Biomédica de Córdoba (IMIBIC), 14004 Avda Menéndez Pidal s/n, Córdoba, Spain; 2 Centro de Investigación Biomédica en Red de Cáncer (CIBERONC), Madrid, Spain; 3 Unidad de Gestión Clínica de Anatomía Patológica, Hospital Universitario Reina Sofía, Córdoba, Spain; 4 Unidad de Gestión Clínica de Cirugía General y del Aparato Digestivo, Hospital Universitario Reina Sofía, Córdoba, Spain; 5 Unidad de Gestión Clínica de Oncología Médica, Hospital Universitario Reina Sofía, Córdoba, Spain; 6 The Institute of Genetics and Molecular Medicine, University of Edinburgh, Western General Hospital, Edinburgh, UK

**Keywords:** Nitric oxide, eNOS, Stem cell, Mesenchymal, *Apc*

## Abstract

**Background:**

Nitric oxide (NO) has been highlighted as an important agent in cancer-related events. Although the inducible nitric oxide synthase (iNOS) isoform has received most attention, recent studies in the literature indicate that the endothelial isoenzyme (eNOS) can also modulate different tumor processes including resistance, angiogenesis, invasion, and metastasis. However, the role of eNOS in cancer stem cell (CSC) biology and mesenchymal tumors is unknown.

**Results:**

Here, we show that eNOS was significantly upregulated in *VilCre*^*ERT2*^*Apc*^*fl/+*^ and *VilCre*^*ERT2*^*Apc*^*fl/fl*^ mouse intestinal tissue, with intense immunostaining in hyperproliferative crypts. Similarly, the more invasive *VilCre*^*ERT2*^*Apc*^*fl/+*^ *Pten*^*fl/+*^ mouse model showed an overexpression of eNOS in intestinal tumors whereas this isoform was not expressed in normal tissue. However, none of the three models showed iNOS expression. Notably, when 40 human colorectal tumors were classified into different clinically relevant molecular subtypes, high eNOS expression was found in the poor relapse-free and overall survival mesenchymal subtype, whereas iNOS was absent. Furthermore, *Apc*^*fl/fl*^ organoids overexpressed eNOS compared with wild-type organoids and NO depletion with the scavenger carboxy-PTIO (c-PTIO) decreased the proliferation and the expression of stem-cell markers, such as *Lgr5*, *Troy*, *Vav3*, and *Slc14a1*, in these intestinal organoids. Moreover, specific NO depletion also decreased the expression of CSC-related proteins in human colorectal cancer cells such as β-catenin and Bmi1, impairing the CSC phenotype. To rule out the contribution of iNOS in this effect, we established an iNOS-knockdown colorectal cancer cell line. NO-depleted cells showed a decreased capacity to form tumors and c-PTIO treatment in vivo showed an antitumoral effect in a xenograft mouse model.

**Conclusion:**

Our data support that eNOS upregulation occurs after *Apc* loss, emerging as an unexpected potential new target in poor-prognosis mesenchymal colorectal tumors, where NO scavenging could represent an interesting therapeutic alternative to targeting the CSC subpopulation.

**Electronic supplementary material:**

The online version of this article (10.1186/s12915-017-0472-5) contains supplementary material, which is available to authorized users.

## Background

Advances in understanding the molecular pathways in colorectal cancer (CRC) have resulted in the development of novel targeted therapeutics [1]. However, CRC remains the third most frequent cancer and the fourth leading cause of cancer deaths worldwide [2]. The cancer stem cell (CSC) model provides an attractive explanation to these mortality data and therapy failure [3]. In the CSC model, a small subpopulation of tumor cells possesses unlimited proliferative potential and chemoresistance, ultimately resulting in a tumor spreading and metastasis [4]. Thus, this cellular subset with tumor-initiating properties has the capacity to evade conventional therapies, which contributes to the adverse survival rates, so new specific targets must be found [5]. Moreover, the heterogeneity of CRC hinders the selection of patients who will respond better to therapy and the detection of new targeted agents [6]. To solve this problem, several gene-expression-based CRC classifications have been reported, dividing the disease into different clinically relevant CRC subtypes [6–10]. Recently, the International CRC Subtyping Consortium analyzed different CRC data sets and have described four robust consensus molecular subtypes (CMSs) associated with clinical variables [11]: CMS1 (microsatellite instability), CMS2 (canonical), CMS3 (metabolic), and CMS4 (mesenchymal). Interestingly, although these classifications differed in the number of tumor subtypes, they all agree in the identification of a stem-like mesenchymal subtype, which is associated with poor patient outcome in CRC [11], so new specific targets must be discovered to improve current treatments.

Nitric oxide (NO) has been highlighted as an important agent in different physiopathological conditions, including colon cancer [12]. It has been shown that NO has a dual role in tumoral processes, being cytotoxic or cytostatic at high levels whereas low levels can have the opposite effect and promote tumor growth [13]. This molecule is synthesized by three different NO synthase (NOS) isoforms using L-arginine and molecular oxygen as substrates: neuronal NOS (nNOS), inducible NOS (iNOS), and endothelial NOS (eNOS) [14]. The inducible isoform has been the center of attention in the study of its role in tumorigenesis [15]. However, there is a huge controversy about the expression and role of iNOS in human colon carcinogenesis. Whereas some authors seem to detect iNOS expression in 60% of human colon adenomas [16], other studies reported that levels of this isoenzyme were low or absent at all stages of colon cancer [17, 18]. Moreover, conflicting results for the role of iNOS in colon tumorigenesis have been found by independent groups using the same *Min* mouse model [19, 20]. On the other hand, recent works in the literature indicate that eNOS may be implicated in different tumor processes, such as resistance to hormonal therapy [21], angiogenesis, invasion, and metastasis [22]. Furthermore, high levels of eNOS have been correlated with an angiogenic phenotype and predict poor prognosis in human gastric cancer [23]. Despite the relation between tumor-expressed eNOS and tumor maintenance [24], there are no data about the role of this protein in the stem-cell-like population responsible for the initiation and maintenance of tumor growth.

In the present study, by employing three different independent mice models, we show that eNOS upregulation is an early event in CRC after *Apc* loss and it is also upregulated in the human mesenchymal CRC subtype. Moreover, we demonstrated that iNOS was absent in all these models as well as in human mesenchymal tumors. We showed that specific removal of eNOS-produced NO with the scavenger carboxy-PTIO (c-PTIO) impaired stem-related signaling pathways essential for CSCs, which decreased in vitro tumorsphere and organoid formation and in vivo tumor formation.

## Results

### eNOS is overexpressed in hyperproliferative regions and tumors of Apc^fl/fl^, Apc^fl/+^, and Apc^fl/+^ PTEN^fl/+^ intestinal mouse tissue

**Fig. 1:**
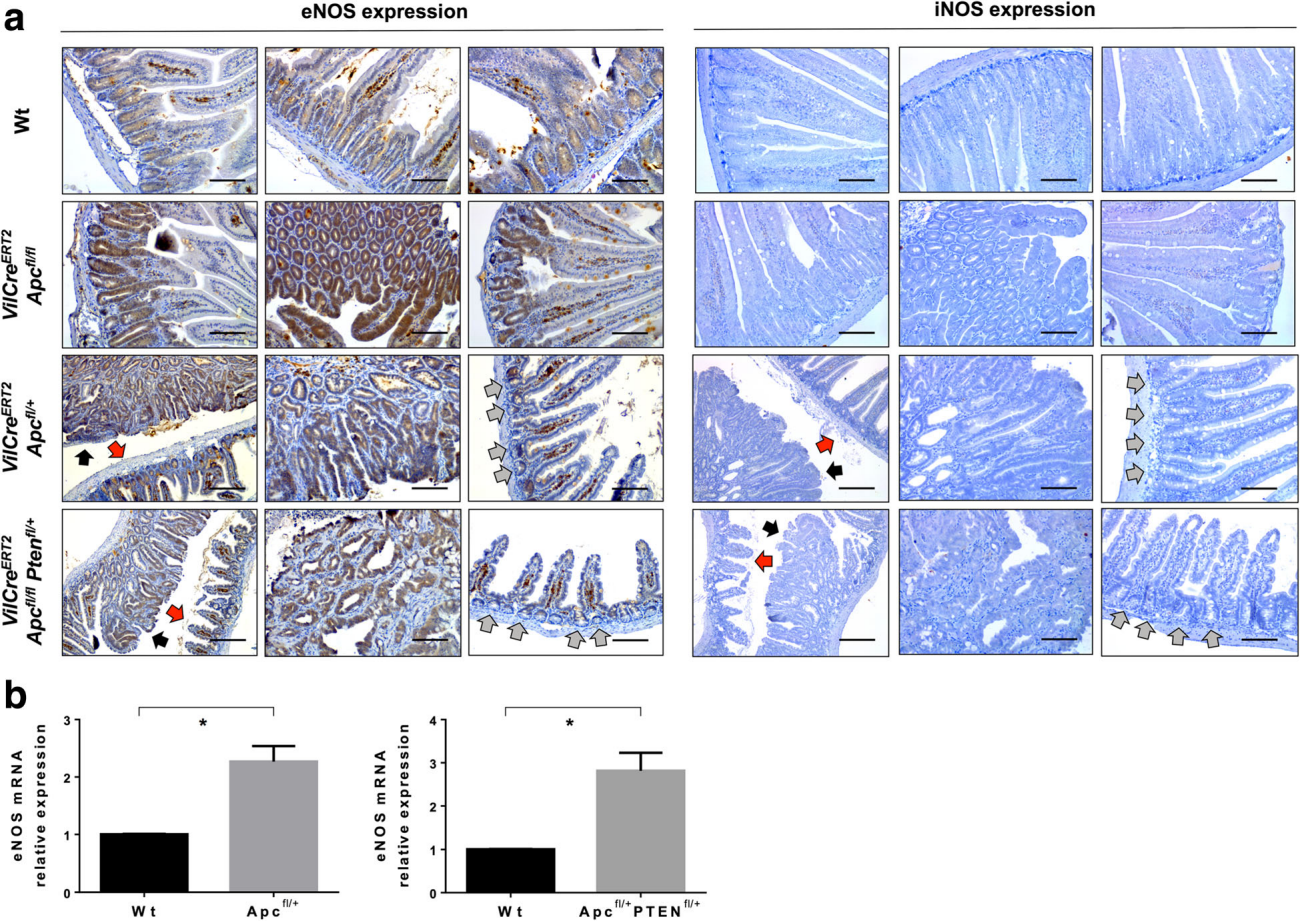
eNOS is upregulated in intestinal tumors from Apc^fl/fl^, Apc^fl/+^, and Apc^fl/+^ Pten^fl/+^ mouse models. **a** eNOS and iNOS immunohistochemistry of wild-type, Apc^fl/fl^, Apc^fl/+^, and Apc^fl/+^ PTEN^fl/fl^ mice intestinal tissues. eNOS expression was absent in the wild-type intestine whereas Apc^fl/fl^ sections showed intense immunostaining in the hyperproliferative crypts. In the Apc^fl/+^ and Apc^fl/+^ PTEN^fl/+^ mice models, hyperproliferative areas resulted in upregulation of eNOS (black arrows) whereas normal intestinal tissue did not show expression of this NOS isoform (red arrow). The bottom of normal crypts did not show staining of eNOS (gray arrows). iNOS expression was absent in all three models. Scale bars: 100 μm and 200 μm. **b** RT-qPCR analysis of eNOS expression from wild-type, Apc^fl/+^ and Apc^fl/+^ PTEN^fl/+^ tissue. * *P* < 0.05 compared with the wild-type non-proliferative tissue. eNOS endothelial NO synthase, iNOS inducible NO synthase, NOS NO synthase, RT-qPCR quantitative real-time reverse-transcriptase polymerase chain reaction, Wt wild type

To explore the role of NO in the generation and maintenance of a CSC subpopulation, we used the CSC-specific *VilCre*^*ERT2*^ *Apc*^*fl/fl*^ mouse model [25], where *Apc*-deficient cells maintain a crypt progenitor-like phenotype associated with the expansion of a stem Lgr5-positive cell population and early colorectal lesions [26]. Whereas intestinal tissue sections from normal mice showed no expression of eNOS, we found intense immunostaining for this NOS isoform in epithelial cells from *Apc*-deficient crypts (Fig. 1a). However, iNOS expression was absent in both wild-type (wt) and *Apc*-deficient intestinal sections (Fig. 1a).

Deletion of both copies of *Apc* leads to a Wnt-driven hyperproliferative phenotype. On the other hand, the conditional deletion of a single copy of *Apc* (*VilCre*^*ERT2*^ *Apc*^*fl/+*^) leads to mice carrying 10–20 discrete adenomas following spontaneous loss of the wt *Apc* allele [27]. An analysis of the tumors that arose in this mouse model showed that an increase of eNOS expression was observed in tumor regions. However, normal areas of the intestine that still remained organized and where tumors were absent did not show any staining at the bottom of intestinal crypts (Fig. 1a). We also found this upregulation of eNOS in intestinal adenomas from *Apc*^*fl/+*^ mouse tissue by RT-qPCR compared with normal intestinal tissue from the same animal (Fig. 1b). The above results suggest that the increase of NO production in mutant crypts through overexpression of eNOS isoenzyme occurs following loss of *Apc*. Again, iNOS was not induced after the loss of just one *Apc* allele (Fig. 1a). Similarly, invasive tumors from the more invasive *VilCre*^*ERT2*^*Apc*^*fl/+*^ *Pten*^*fl/+*^ mouse model showed an overexpression of eNOS in tumors whereas normal crypts did not show any expression of this isoenzyme (Fig. 1a). Accordingly, RT-qPCR analyses confirmed that eNOS was almost threefold upregulated in the intestine of *VilCre*^*ERT2*^*Apc*^*fl/+*^ *Pten*^*fl/+*^ mice compared with normal tissues (Fig. 1b). As found in the other two mouse models, iNOS expression was absent in both tumor and normal epithelium adjacent to the tumor (Fig. 1a).

Therefore, the above results suggest that there may be a relevant role for the upregulation of eNOS expression in the early stages of CRC, where the Lgr5-positive stem cell population is in expansion, and also that the upregulation of this NOS isoform is maintained in more advanced and invasive genotypes.

### eNOS is upregulated in the mesenchymal poor-prognosis subtype and poorly differentiated human colorectal tumors

We next evaluated the expression of NOS isoforms in different CRC subtypes. We classified 40 human colorectal adenocarcinomas according to Sadanandam et al. [10] and De Sousa et al. [6]. Thus, we obtained a supervised classification of tumors into five molecularly distinct subtypes, which are associated with different clinically relevant characteristics: transit-amplifying, enterocyte, goblet-like, inflammatory, and stem-cell-like (Additional file 1: Figure S1 and Additional file 2: Figure S2).

**Fig. 2:**
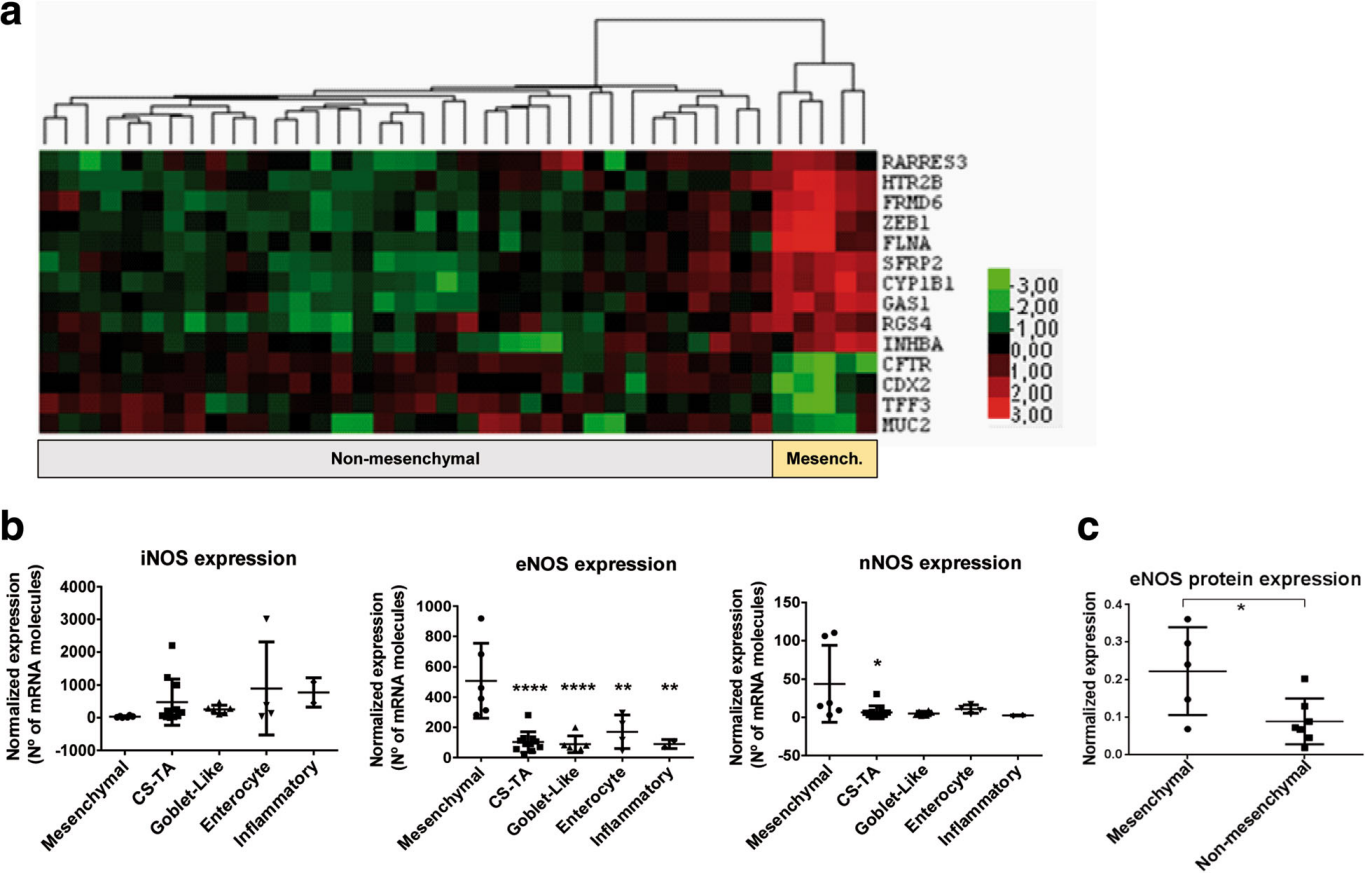
eNOS is significantly upregulated in human mesenchymal CRC tumors. **a** Unsupervised classification of the mesenchymal subgroup of CRC tumors and non-mesenchymal subtypes. The heat map was generated using nSolver software from NanoString Technologies. **b** eNOS expression as number of mRNA molecules detected. Te plots show normalized data of eNOS gene expression in all human CRC subtypes using the nCounter system from NanoString Technologies. **c** eNOS Western blot analysis of total protein extracts of classified human CRC tumors. *P < 0.05, **P < 0.01, ****P < 0.0001 compared with non-mesenchymal tumors. CRC: colorectal cancer, eNOS: endothelial NO synthase, iNOS: inducible NO synthase, CS-TA: colorectal subtype transit-amplifying

**Fig. 3:**
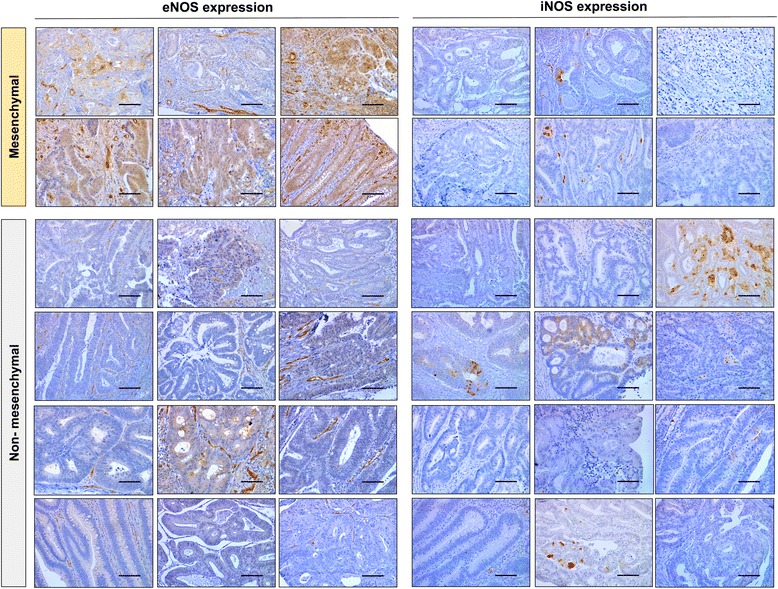
eNOS and iNOS immunohistochemistry in classified human CRC tumors. eNOS is highly expressed in human mesenchymal CRC tumors. Mesenchymal tumors showed a high expression of eNOS whereas this isoenzyme was low or absent in non-mesenchymal tumors. iNOS expression was found sporadically among the tumors and was restricted to particular areas or even individual cells where the induction may be triggered. Scale bars: 100 μm. CRC: colorectal cancer, eNOS: endothelial NO synthase, iNOS: inducible NO synthase

The unsupervised classification (Fig. 2a) confirmed the identification of this stem-like subgroup of tumors that match the mesenchymal CMS4 group recently defined by the International CRC Subtyping Consortium [11]. We also analyzed the expression of cancer-related human miRNAs in different classified tumors and we found a significant signature of 11 miRNAs, which defines the mesenchymal subtype compared to the other tumor subtypes (Additional file 3: Figure S3a). The overexpression of miR-100, let-7e, and miR-99a, which have been shown to be powerful regulators of the epithelial-to-mesenchymal transition (EMT) [28–30], was found in the mesenchymal tumor subtype. However, miR-215 [31], miR-375 [32], miR-141, and miR-200c [33], miR-200a [34], miR-429 [35], miR-625 [36], and miR-18a [37] have already been shown to be inversely correlated with the EMT, and they were found downregulated in this subtype. Similarly, we obtained a specific immune response in the mesenchymal CRC subtype (Additional file 3: Figure S3b), for which a significant increase of innate immune cells was found (Additional file 4: Figure S4a) as well as an upregulation of immune response genes (Additional file 4: Figure S4b). All three signatures generate a robust classification of the mesenchymal tumors, which differentiate them from the other tumors. Notably, the analysis of NOS isoforms showed that, compared to the other non-mesenchymal CRC subtypes, eNOS was highly expressed in the mesenchymal subtype whereas iNOS was nearly absent (Fig. 2b). In agreement with RNA expression analyses, protein expression (Fig. 2c) and immunohistochemical studies (Fig. 3) confirmed the higher expression of eNOS protein in mesenchymal tumors compared to the other non-mesenchymal CRC subtypes.

The expression of iNOS was found in some human colorectal tumors and it was restricted to just some aberrant crypts or even individual cells, unlike the general high expression of eNOS in mesenchymal tumors (Fig. 3). The induction of iNOS occurs in punctual regions where proper inductive signals may lead to a massive increase in its expression. This produces just temporary high levels of NO, since this is energetically unsustainable over time. Interestingly, immunohistochemical analyses of unclassified high-grade poorly differentiated tumors from CRC patients also showed the upregulation of eNOS compared with non-proliferative areas (Additional file 5: Figure S5). In line with these results, a significantly decrease was found in 5-year survival in patients with CRC, colon adenocarcinoma, and rectum adenocarcinoma when eNOS was upregulated. However, a low expression of iNOS isoform decreased the 5-year survival of these patients (Additional file 6: Figure S6). Thus, as found for mesenchymal CRC tumors, the overexpression of eNOS and low expression of iNOS resulted in a poor prognosis in CRC. Overall, the above results suggest that the eNOS isoform is the main source of NO in mesenchymal poor-prognosis tumors.

### eNOS is upregulated in intestinal epithelial organoids derived from Apc^fl/fl^ mice and NO scavenging decreases their proliferation and stem-cell marker expression

**Fig. 4:**
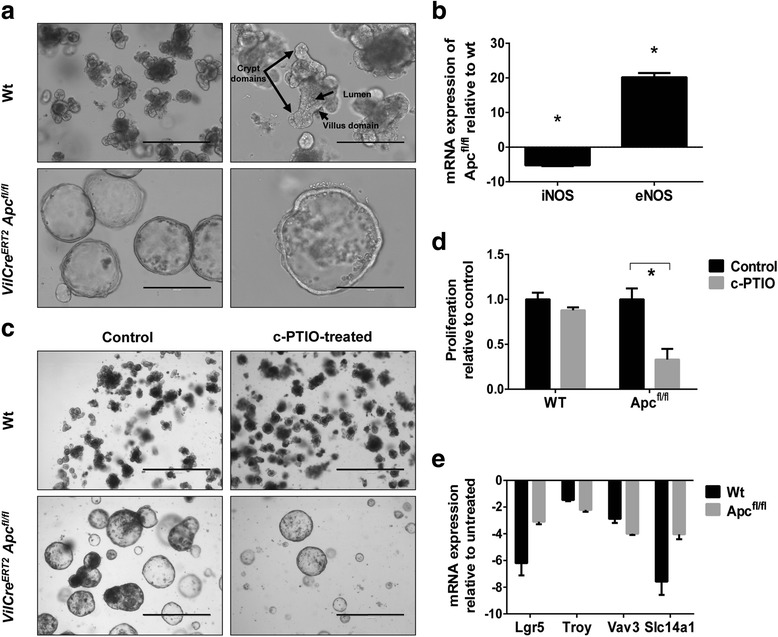
eNOS is upregulated in organoids derived from Apc^fl/fl^ mice intestine and NO trapping decreases their proliferation and downregulates the expression of stem-cell markers. **a** Representative images from typical wild-type and Apc^fl/fl^ organoids. Scale bars: 400 μm (left) and 200 μm (right). **b** RT-qPCR analysis of iNOS and eNOS expression from wild-type and Apc^fl/fl^ organoids. *P < 0.05 compared with the wild-type organoids. **c** and **d** Proliferation assay of control or wild-type and Apc^fl/fl^ organoids treated for 48 hours with c-PTIO (500 μM). Proliferation was determined by image analysis with ImageJ software. *P < 0.05 compared with the control. Scale bars: 1000 μm. **e** RT-qPCR analysis of Lgr5, Troy, Vav3, and Slc14a1 expression from control or c-PTIO (500 μM) treated wild-type and Apc^fl/fl^ organoids. eNOS endothelial NO synthase, iNOS inducible NO synthase, RT-qPCR quantitative real-time reverse-transcriptase polymerase chain reaction, WT (wt, Wt) wild-type

Intestinal epithelial cells from wt or tamoxifen-induced *Apc*^*fl/fl*^ mice were cultured using the organoid-forming assay and, whereas wt organoids grew to form crypt-like structures, *Apc*^*fl/fl*^ organoids completely lost their intestinal structure (Fig. 4a). Analysis of expression of NOS isoforms showed that eNOS was highly expressed in *Apc*-deficient organoids compared to their normal counterparts, whereas iNOS was downregulated (Fig. 4b). This result supports again that the eNOS isoform could have an important role during the expansion of the Lgr5-positive stem-cell subpopulation after *Apc* loss. Scavenging of NO with c-PTIO, a compound that quickly reacts with NO decreasing its concentration and bioavailability for tumor cells, caused a significant decrease of proliferation of *Apc*^*fl/fl*^ organoids whereas wt organoids grew normally (Fig. 4c,d). Moreover, NO depletion also caused a marked decrease in the Lgr5^+^ cell population both in wt and in *Apc*^*fl/fl*^ organoids (Fig. 4e). Furthermore, other stem-cell markers, including *Troy*, *Vav3*, and *Slc14a1*, were downregulated after NO scavenging (Fig. 4e). Thus, these results support again an important regulatory role of NO in intestinal CSC biology.

### NO depletion abolishes the capacity of CRC cells to form tumorspheres and organoids in vitro and impairs CSC-related pathways

**Fig. 5:**
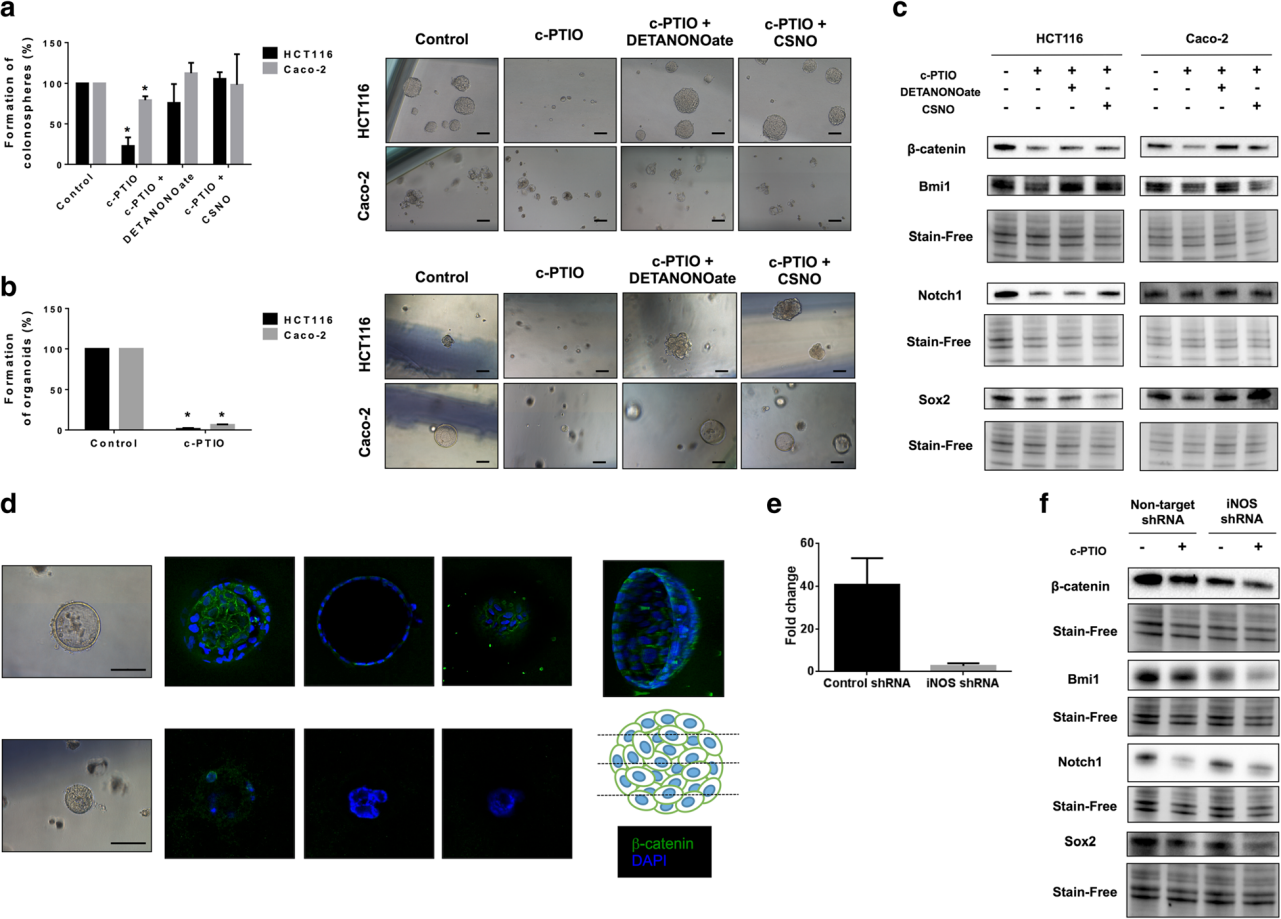
eNOS-derived NO scavenging significantly impairs the CSC phenotype in human colon cancer cells. **a** Formation of tumorspheres from HCT-116 and Caco-2 cells pre-treated for 24 hours with c-PTIO (100 μM) with or without DETANONOate or CSNO (100 μM). Representative images 1–2 weeks after seeding are shown. **b** Formation of organoids from cells pre-treated for 24 hours with c-PTIO (100 μM) with or without NO donors (100 μM). Representative images 1–2 weeks after seeding are shown. **c** Immunoblot of β-catenin, Bmi1, Notch1, and Sox2 expression in HCT-116 and Caco-2 tumor cells treated for 24 hours with c-PTIO (100 μM) with or without DETANONOate or CSNO (100 μM). **d** Organoid immunofluorescence of β-catenin from control or Caco-2 organoids treated with c-PTIO (100 μM) for 24 hours. **e** RT-qPCR of iNOS-induced expression (IL-1β 3 ng/ml, IFN-γ 200 U/ml and TNF-α 75 ng/ml, for 6 hours) from control and iNOS-shRNA expressing HCT-116 cells. **f** Immunoblot of previously mentioned proteins in iNOS-knockdown HCT-116 cancer cells treated with c-PTIO (100 μM). Stain-free technology was used as loading control in immunoblot experiments. * *P* < 0.05 compared with the control. Scale bars: 100 μm. CSC cancer stem cell, CSNO S-nitrosocysteine, eNOS endothelial NO synthase, iNOS inducible NO synthase, RT-qPCR quantitative real-time reverse-transcriptase polymerase chain reaction

To study the role of NO in the CSC subpopulation, we performed tumorsphere experiments to characterize the CSC subpopulation in vitro. This is a functional assay of self-renewal capacity. We found that the pre-treatment of HCT-116 and Caco-2 cells with c-PTIO markedly inhibited their subsequent ability to form tumorspheres (Fig. 5a). This effect was NO-specific since the addition of NO donors, such as DETANONOate or S-nitrosocysteine (CSNO), eradicated the anti-CSC activity of c-PTIO in HCT-116 and Caco-2 cells (Fig. 5a). Thus, these results suggest an important regulatory role of NO in the self-renewal capacity of colorectal CSCs. On the other hand, the specific depletion of NO in HCT-116 and Caco-2 cells led to a large decrease in the size and number of organoids formed (Fig. 5b and Additional file 7: Figure S7). This was a NO-specific effect since the use of NO donors recovered the capacity of HCT-116 and Caco-2 cells to form organoids (Fig. 5b). From these results, NO scavenging downregulated the levels of β-catenin, Bmi1, Notch1, and Sox2 in HCT-116 and Caco-2 cells. The use of NO donors showed again the capacity to modulate key CSC signaling proteins depending on the levels of this molecule (Fig. 5c). We also performed a confocal immunofluorescence of β-catenin in the *Apc* mutated Caco-2 organoids [38] and found a downregulation of this protein after NO scavenging (Fig. 5d). To rule out the contribution of iNOS in this effect, we developed iNOS-knockdown HCT-116 cells (Fig. 5e). Scavenging NO in this iNOS-deficient background showed the downregulation of all the mentioned proteins (Fig. 5f), highlighting the relevance of eNOS-produced NO.

The above results demonstrate a regulatory role for NO in the CSC phenotype and emphasize the importance of the production of this molecule by eNOS in maintaining this phenotype. Moreover, NO scavenging is shown to be an appealing alternative to the typically used iNOS inhibitors in cancer, particularly in stem-cell-like or mesenchymal tumors.

### NO scavenging reduces the capacity of mesenchymal CRC cancer cells to form tumors in xenograft mouse models

**Fig. 6:**
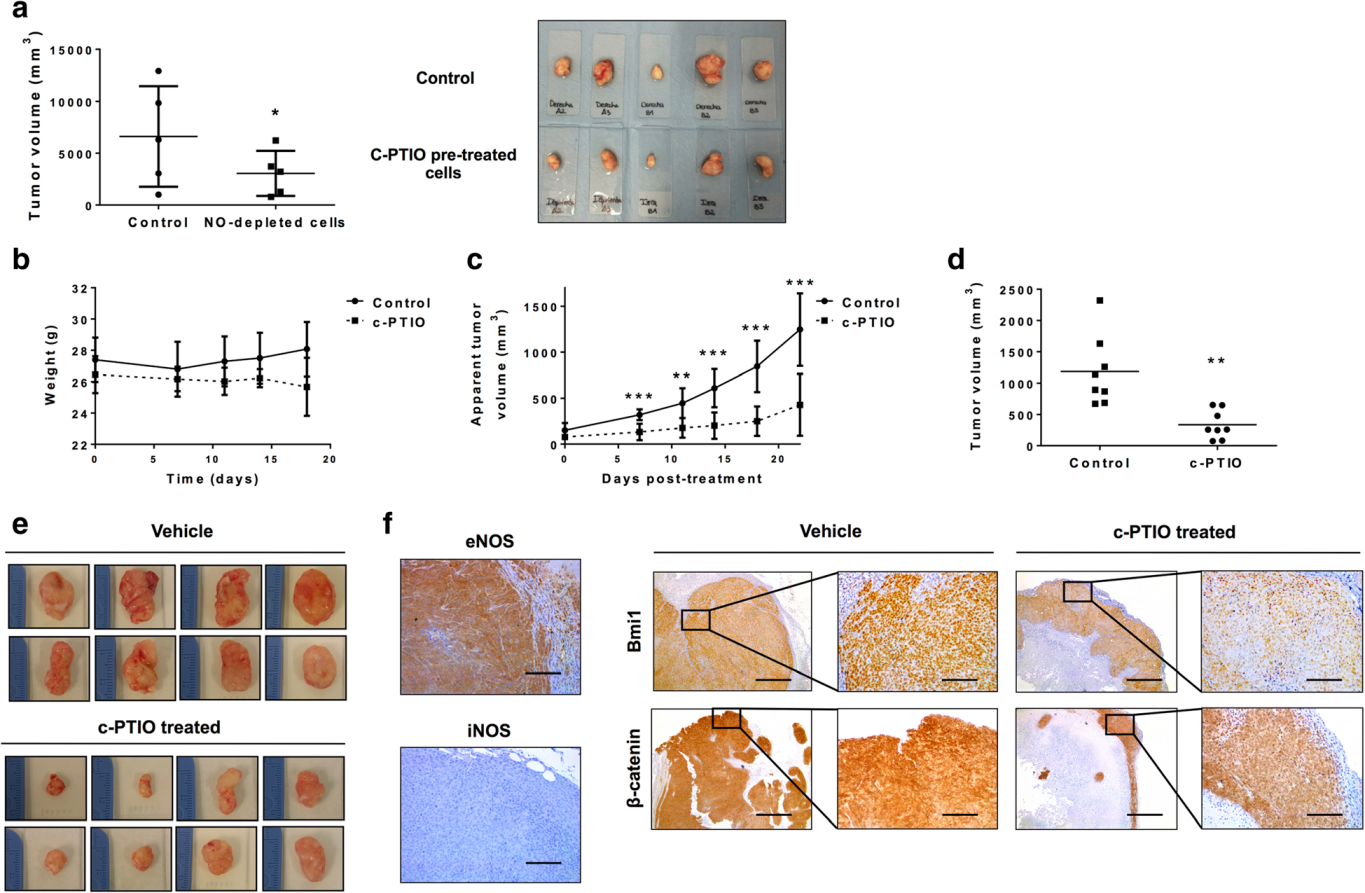
NO trapping with c-PTIO reduces the capacity of mesenchymal HCT-116 cells to form tumors in xenografted mice and decreases expression in vivo of β-catenin and Bmi1. **a** Control or HCT-116 cells pre-treated with c-PTIO (100 μM) for 24 hours were subcutaneously injected into the flanks of immunocompromized mice. Final tumor volumes were analyzed after 37 days of inoculation. Mice xenografted with HCT-116 cells were treated with vehicle or c-PTIO (320 mg/kg) once a week. **b** Mice weights were monitored over time. **c** Tumor size was measured during the course of treatment. **d** and **e** Final tumor volume was analyzed after harvesting. **f** Representative images from immunohistochemistry of eNOS and iNOS from HCT-116-derived tumors and β-catenin and Bmi1 from vehicle or c-PTIO treated mice tumors. *P < 0.05, **P < 0.01, ***P < 0.001 compared with control. eNOS endothelial NO synthase, iNOS inducible NO synthase

To explore the antitumor capacity of NO depletion further, two different xenograft experiments were performed using mesenchymal HCT-116 CRC cells [10]. Thus, control and c-PTIO-treated HCT-116 cells were subcutaneously injected into immunocompromized mice and then tumor progression was evaluated. As shown in Fig. 6a, NO-depleted cells generated tumors half the size of those generated from untreated control cells. The decreased capacity of c-PTIO pre-treated cancer cells to form larger tumors suggest a long-term effect of scavenging NO and an important role of this molecule in the regulation of CSCs. On the other hand, to elucidate the antitumor effectiveness of c-PTIO in vivo, HCT-116 cells were subcutaneously injected into immunocompromized mice and animals were then treated with 320 mg/kg of c-PTIO once a week. The treatment had no toxic effects and did not cause weight loss during the experiment (Fig. 6b). In vivo c-PTIO treatment attenuated tumor growth (Fig. 6c) and final tumor volume was significantly decreased compared with that of untreated control mice (Fig. 6d,e). Interestingly, tumor xenografts did not show any expression of iNOS whereas eNOS was found highly expressed in both epithelial and endothelial cells (Fig. 6f). Thus, the effect of c-PTIO was the result of eNOS-produced NO scavenging, the main source of this molecule in a mesenchymal tumor. Moreover, the expression of β-catenin and the relevant target for cancer therapeutics, Bmi1 [39], were found decreased in tumors after c-PTIO treatment (Fig. 6). Again, these results further support a key role of eNOS-derived NO in CSC maintenance and show NO scavenging as an attractive alternative for targeting this tumor subpopulation.

## Discussion

Although there is emerging evidence indicating that CSCs are responsible for cancer aggressiveness, chemoresistance, and relapse, which results in high mortality rates [40], there is a little knowledge about their regulation. Different biological mediators in tumor microenvironments may cause reversible changes in a CSC population [41]. The iNOS isoform has been shown to be the predominant enzyme that promotes tumor progression through NO production [42] and has prompted the search for new therapies based on inhibitors against the iNOS isoform [43]. Nevertheless, there is a huge controversy about the role of iNOS in colon cancer [16–20], and this approach offers a limited therapeutic benefit in contexts where constitutive isoforms are the main or even the only source of NO [44]. Moreover, there are non-enzymatic sources of NO, which are not blocked by NOS inhibitors [45]. Furthermore, NO scavenging shows a second-order reaction kinetic, decreasing NO preferentially in regions of aberrant overproduction and having a light impact on essential basal NO levels [46]. Thus, NO scavengers emerge as an alternative approach to targeting this molecule before it can exert its effects, trapping NO from all sources.

In 1997, Takahashi et al. showed there was increased expression of eNOS in endothelial cells of azoxymethane-induced rat colon tumors [47]. Although the effects of NO from the eNOS isoenzyme on tumorigenesis have been mainly related to its expression in endothelial cells [48], tumor-expressed eNOS and its role in initiation and maintenance of the tumorigenic process have been previously shown in other tumors [24]. Actually, an upregulation of eNOS expression was found in undifferentiated regions compared to differentiated tumor areas in a murine mammary tumor model, and strongly eNOS-positive tumor cells have been found in lung metastatic sites [44].

Here, to elucidate the role of NO in CSC biology, we used the early tumorigenesis *VilCre*^*ERT2*^ *Apc*^*fl/fl*^ mouse model, where the deletion of both copies of this gene results in a crypt progenitor-like phenotype characterized by augmented proliferation and CSC subset expansion [49]. Epithelial cells from intestinal *Apc*^*fl/fl*^ crypts showed intense immunostaining of eNOS, whereas crypts from wt mice did not show any expression of this isoenzyme. In fact, the conditional *VilCre*^*ERT2*^ *Apc*^*fl/+*^ mouse model showed that the haploinsufficiency of the *Apc* gene is enough to upregulate the expression of eNOS and increase the production of NO in tumors. These results support the relevance of eNOS isoform in this context of Lgr5-positive stem cell expansion. The production of NO in the colon has been linked with a higher risk of colon cancer [50] and our results suggest that the eNOS isoenzyme seems to be an important source of these molecules in early mutational events of the disease. Similarly, invasive tumors from the more invasive *VilCre*^*ERT2*^*Apc*^*fl/+*^ *Pten*^*fl/+*^ mouse model showed that eNOS was upregulated in tumors whereas normal crypts did not show any expression. Interestingly, iNOS expression was absent in all three independent mouse models, supporting the hypothesis that eNOS is the most relevant NOS during the first stages of the disease.

We also classified 40 human colorectal tumors into five molecular subtypes according to Sadanandam et al. [10] and De Sousa et al. [6]. The stem-like subtype matches the mesenchymal CMS group of the most robust classification system currently available for CRC and has the worst relapse-free and overall survival [11]. This mesenchymal phenotype has been shown to be regulated by different pleiotropic-acting molecules, including miRNAs [51]. Here, we obtained a significant signature of 11 miRNAs, which differentiates the mesenchymal subtype from the other tumors. Similarly, we obtained a specific immune response in the classified human mesenchymal tumors. In particular, a significant increase of dendritic cells, CD45 cells, macrophages, and mast cells was found in mesenchymal tumors. This is consistent with the fact that the stromata of these tumors are infiltrated with endothelial cells, cancer-associated fibroblasts, and also innate immune cells [11, 52]. Moreover, these tumors showed an upregulation of immune response genes such as complement signaling genes, whose overexpression has been shown in mesenchymal tumors [11]. These three signatures generate a robust classification of the mesenchymal tumors, which differentiates them from the other tumors. Notably, gene expression analysis of NOS isoforms revealed that eNOS was significantly upregulated in the mesenchymal subtype whereas iNOS was absent. This result suggests that eNOS is the most relevant enzymatic source of NO in this poor-prognosis tumor subtype and may represent an active stem-cell regulatory point in cancer as well as a possible target for therapy against aggressive human tumors.

In vitro tumor organoid models could be used to interrogate the sensitivity toward different drugs and to predict patient response [53]. Here, we found that eNOS was significantly upregulated in *Apc*^*fl/fl*^ organoids compared to their normal counterparts whereas iNOS was downregulated. Moreover, we showed that scavenging NO with the highly specific NO scavenger c-PTIO [54] significantly decreased the proliferation of *Apc*-deleted organoids, markedly decreasing the Lgr5 population and downregulating the expression of other stem-cell markers including *Troy* [55], *Vav3* [56], and *Slc14a1* [57]. These results support again the implication of NO in maintaining the CSC phenotype. Moreover, the withdrawal of NO with c-PTIO impaired the CSC phenotype of CRC cells, decreasing their self-renewal capacity and the expression of key proteins considered as putative targets in CSC signaling pathways, such as Notch, β-catenin, and Bmi1 [3]. The effect was molecule-specific since the use of NO donors allowed tumor cells to recover their initial phenotype and abrogated the anti-CSC effect of c-PTIO. Furthermore, scavenging NO with c-PTIO in iNOS-knockdown HCT-116 cells also resulted in an impairment of the CSC phenotype, highlighting the relevance of eNOS-produced NO. These results suggest that targeting CSC self-renewal through NO scavenging could be a valuable approach in cancer therapy.

According to the CSC model, only this subset of tumor cells has the capacity to generate tumors in xenotransplantation experiments with immunocompromized mice due to its self-renewal properties and wide proliferative potential [58]. Herein, the abrogation of NO for 24 hours generated tumors with 50% less volume in a xenograft model with mesenchymal HCT-116 CRC cells. This result supports the idea that NO trapping causes long-term changes in the CSC phenotype of tumor cells, decreasing their enormous proliferative capacity. Furthermore, we found an impaired tumor growth capacity in c-PTIO-treated mice, which formed tumors significantly smaller than those of untreated mice. These tumors did not show iNOS expression, whereas intense immunostaining of eNOS was found. Moreover, tumors formed in c-PTIO-treated animals showed a downregulation of β-catenin and Bmi1 expression, which is a key protein required for CSC function in colorectal tumors and represents an effective target for controlling tumor growth [59].

## Conclusion

In summary, our data show that eNOS upregulation is as an early event in CRC together with *Apc* loss and this overexpression is maintained in other more advanced tumor genotypes. This isoenzyme is upregulated in different scenarios where the CSC phenotype is enhanced, including different mice conditional CRC models, human mesenchymal CMS tumors, and poorly differentiated aggressive adenocarcinomas. Moreover, the NO scavenger c-PTIO has been shown to impair the stem-related signaling pathways essential for CSCs in vitro and to have an antiproliferative effect in vivo, decreasing β-catenin and Bmi1 expression in a xenograft mouse model. Since targeting the CSC population is increasingly becoming a priority in cancer research, our results indicate that eNOS is an unexpected potential new target in human poor-prognosis mesenchymal colorectal tumors.

## Methods

### Patients and inclusion criteria

Forty patients over 18 years of age with resectable CRC and submitted to surgery in Reina Sofía Hospital (Córdoba, Spain) were included in the study, which was approved by the Reina Sofía Hospital ethical committee. Signed informed consent was obtained from each patient.

### Classification into colon cancer subtypes

We used the nCounter Element system from NanoString to analyze the RNA expression of a set of genes and classify CRC samples according to Sadanandam et al. [10] (five subtypes) and De Sousa et al. [6] (three subtypes), both classifiers strongly relating to each other. Data analysis was performed using nSolver software (NanoString Technologies, Seattle, WA, USA) to manage the raw data generated for the expression of each gene. Then, the raw data expression readouts were normalized and an overall average (mean) was established for each gene. The positive expression of one particular gene indicates that the number of RNA molecules is above the average, whereas negative expression indicates that it is below the mean of the number of RNA molecules. An miRNA signature from different tumor subtypes was obtained using the nCounter human v3 miRNA expression assay from NanoString. Tumor immune response in the different tumor subtypes was obtained using the nCounter PanCancer immune-profiling panel from NanoString.

### Immunohistochemistry

First, 4-μm-thick sections were mounted on poly-L-lysine coated slides, deparaffinized in xylene, and rehydrated using graded alcohols. Antigen retrieval was accomplished with a 10 mM citrate buffer (pH 6.0) at 95–98 °C for 20 min. Endogenous peroxidase was neutralized using EnVision FLEX peroxidase-blocking reagent (Dako, Denmark) for 10 min. Tween-phosphate-buffered saline (PBS) was used as the washing solution. Tissue sections were blocked with 3% bovine serum albumin and the mouse-on-mouse staining protocol (Abcam) was used for mouse samples with eNOS mouse-produced antibody. Anti-eNOS (6H2) (1:200, Cell Signaling, Danvers, MA, USA, ref 02/2016, lot 2, RRID AB_10850618), anti-iNOS (1:100, Invitrogen, lot RJ2276825, RRID AB_2537941), anti-Bmi1 (1:400, Abcam, lot GR301384-1, RRID AB_2065390), and β-catenin (BD, East Rutherford, NJ, USA, 1:100, lot 5121508, RRID AB_397554) were used as primary antibodies. EnVision FLEX + mouse (linker) (Dako) was use for eNOS staining in mouse samples. EnVision FLEX/HRP (Dako K8000) was used as the secondary antibody for 30 min at room temperature, followed by 3,3'-diaminobenzidine (DAB) staining (Dako liquid DAB + substrate chromogen system, Dako). Sections were then counterstained with hematoxylin, dehydrated, and mounted.

### Quantitative real-time reverse-transcriptase polymerase chain reaction

Total RNA extraction was performed using RNeasy Mini Kit (Quiagen, Hilden, Germany) following the manufacturer’s recommendations. iScript gDNA clear cDNA synthesis kit (Bio-Rad) was used to obtain cDNA and real-time PCR was performed using Sensifast SYBR NO-ROX mix (Bioline, London, UK). Expression levels of *eNOS*, *iNOS*, *Lgr5*, *Troy*, *Vav3*, and *Slc14a1* were measured by quantitative real-time PCR (qPCR) using LightCycler 480 Instrument II (Roche, Basel, Switzerland). Primer sequences are described in Additional file 8: Table S1. Results were normalized to glyceraldehyde 3-phosphate dehydrogenase (GADPH) and relative expression was determined by the 2^-∆∆Ct^ method.

### Chemicals

c-PTIO was purchased from Sigma-Aldrich (St Louis, MO, USA) and DETANONOate from Cayman Chemical (Ann Arbor, MI, USA). S-nitrosocysteine (CSNO) was synthesized as previously described [60] by incubation of L-cysteine with acidified sodium nitrite and quantification by absorbance at 334 nm using a molar absorption coefficient of 0.74/mM/cm. IL-1β, IFN-γ, and TNF-α were purchased from R&D Systems.

#### Cell cultures

HCT-116 cells (DSMZ, Braunschweig, Germany) were grown in McCoy’s 5A medium (Biowest) containing 10% fetal bovine serum (FBS) (PAA Laboratories, Pasching, Austria). Caco-2 cells (ECACC, Salisbury, UK) were grown in MEM medium (Biowest, Nuaillé, France) containing 15% FBS (PAA Laboratories) and MEM non-essential amino acids (Biowest). Culture media were supplemented with 2 mM glutamine (Biowest) and Zell Shield antibiotics (Minerva Biolabs, Berlin, Germany). Cells were maintained in a humidified atmosphere at 37 °C and 5 °C CO_2_.

### Western blot analysis

Cells were treated with c-PTIO, c-PTIO and DETANONOate, or CSNO for 24 hours and protein lysate extraction and immunoblotting were performed as described elsewhere [61]. Primary antibodies recognizing Notch1 (Abcam, Cambridge, UK, 1:2000, lot GR215257-19, RRID AB_881725), Bmi1 (Abcam, 1:5000), β-catenin (BD, 1:2000) and Sox2 (R&D Systems, Minneapolis, MN, USA 1:1000, lot KOY0212101, RRID AB_355110) were used. Stain-free technology (Bio-Rad, Hércules, CA, USA) was used as loading protein control and densitometric analysis was performed with Image Lab software (Bio-Rad).

### Colonosphere formation assay

Cells were treated while growing in adherence and then trypsinized and seeded at clonal density (1 cell/μL) in ultra-low attachment surface 96-well plates (Costar, Corning, NY, USA) with serum free Dulbecco’s MEM Nutrient Mixture F + 12 HAM (Sigma-Aldrich) supplemented with 1× B27 (Invitrogen, Carlsbad, CA, USA), 10 ng/ml bFGF (Prepro Tech, London, UK), 20 ng/ml EGF (Santa Cruz Biotechnology, Heidelberg, Germany) and 1% (v/v) methylcellulose (R&D Systems) to prevent cell aggregation. Every 2–3 days, freshly supplements were added. The number and size of colonospheres were analyzed by optical microscopy 1–2 weeks after seeding.

### Organoid formation assay

Cells were treated while growing in adherence and then trypsinized and embedded in Matrigel (growth factor reduced, phenol red free; BD) on ice and seeded in 24-well plates (10–25 single cells/μL of Matrigel per well). The Matrigel was polymerized for 10–15 min at 37 °C and then 500 μL/well basal culture medium (advanced DMEM/F12) supplemented with Zell Shield antibiotics (Minerva Biolabs), 1× B27 and 1× N2 (Thermo Scientific, Waltham, MA, USA) was added. The number and size of organoids were analyzed by optical microscopy 1–2 weeks after seeding.

For wt or *VilCre*^*ERT2*^*Apc*^*fl/fl*^ mouse organoids, crypts, fragments of epithelium, or single cells were seeded in 24-well plates (50 and 10 fragments per 10 μL of Matrigel per well, respectively). After 24 hours, organoids were treated with c-PTIO for 48 hours and proliferation was determined by analyzing the images with ImageJ software.

### Confocal immunofluorescence: in situ staining of organoids within Matrigel

Cells were plated in 60 μL of Matrigel in μ-slide eight-well ibiTreat chamber slides (Ibidi, Martinsried, Germany) and overlaid with advanced DMEM/F12 supplemented with Zell Shield antibiotics, 1× B27 and 1× N2 supplement. Cell-derived organoids were observed for 16 days and then they were fixed in situ for 5 min each with −20 °C 100% methanol and 2% formaldehyde in PBS. The chambers were washed twice for 5 min with PBS and once for 5 min with PBS-Tween. The slides were blocked with 3% bovine serum albumin for 30 min and incubated with β-catenin antibody (BD, 1:50) at 4 °C overnight and washed three times for 5 min with PBS-Tween. Organoids were then incubated with Alexa Fluor 488 secondary antibody (Thermo Fisher, 1:200) at 4 °C overnight and were washed three times for 5 min with PBS-Tween. Nuclei were stained with DAPI and serial images were taken using a Zeiss LSM 710 confocal microscope.

### Lentiviral particles transduction and iNOS-silenced clone selection

HCT-116 cells were seeded (12,000 cells/well) in 96-well plates to be transduced at 50–80% confluency. To enhance the transduction, 8 μg/ml hexadimethrine bromide (Sigma-Aldrich) was added to the cells. Then, viral particles containing iNOS shRNA or non-target control shRNA were added at 5× multiplicity of infection and the cell–viral particle mixture was incubated at 37 °C overnight. After incubation, the viral-particle-containing medium was removed and replaced with fresh complete culture medium. Cells were expanded to obtain a sufficient amount for selection of transduced cells. Then, cells were harvested in filtered (0.2 μm) basic sorting buffered (1× phosphate buffer, 2 mM EDTA, 25 mM HEPES pH 7.0, 2% FBS, and Zell Shield antibiotics) and green fluorescent protein-positive cells were sorted by flow cytometry (FACSAria III, BD) with a 100-μm nozzle in a cloning 96-well plate. Clones were grown in 50% FBS complete medium and iNOS downregulation was confirmed by RT-qPCR.

### Mouse xenograft model

The experiment with mice was approved by the Instituto Maimónides de Investigación Biomédica de Córdoba (IMIBIC) Ethical Committee and followed all the ethical protocols established. To establish a subcutaneous colorectal xenograft model, control cells or cells treated with c-PTIO for 24 hours (2.5 × 10^6^ HCT-116 in 100 μL Matrigel) were implanted subcutaneously into the flanks of 5-week-old immunocompromized male NOD/SCID mice (NOD.CB17/AlhnRj-Prkdc^scid^, Janvier Labs). The experiment was terminated 37 days post injection and tumor volume was analyzed. To evaluate the in vivo effectiveness of c-PTIO, both hind flanks of 6-week-old male NOD/SCID mice (NOD.CB17/AlhnRj-Prkdc^scid^, Janvier Labs) were injected subcutaneously with 4 × 10^6^ HCT-116 cells in 100 μL Matrigel. Eight-week-old mice were treated with one intraperitoneal injection per week of c-PTIO (320 mg/kg) or vehicle (PBS). Tumor growth was measured weekly using a digital caliper. The apparent tumor volume was calculated as *lw*^2^/2 and final tumor volume as (π/6) × *lw* ×*h*  (*l*:lenght; *w*:width; *h*:height).

#### Statistical analysis

Student’s test was used to test for statistical significance. Error bars represent mean ± standard deviation.

## Additional files

Additional file 1: Figure S1. Qualitative supervised classification of human CRC tumors into different CRC subtypes. Data analysis was performed using nSolver software from NanoString Technologies. Raw expression data were normalized and an overall average was established for each classifier gene, as described by Sadanandam et al. [10] and De Sousa et al. [6]. Positive expression indicates a value of expression above the average and it is shown as a green square, whereas negative expression is a value below the overall mean and it is shown as a red square. (TIF 64176 kb)

Additional file 2: Figure S2. Relative expression of classifier genes in all tumor samples. List of classifiers genes used and their relative expression in all tumor samples: mesenchymal, transit-amplifying, goblet-like, enterocyte, and inflammatory. Data analysis was performed as described in “Methods.” (TIF 51415 kb)

Additional file 3: Figure S3. The human mesenchymal CRC subtype has a specific miRNA and tumor immune response signature. Heat maps were generated using nSolver software from NanoString Technologies. **a** miRNA signature of mesenchymal tumors compared with other non-mesenchymal subtypes. **b** Tumor immune response signature of mesenchymal tumors compared with non-mesenchymal tumors. (TIF 59213 kb)

Additional file 4: Figure S4. NanoString immune-profiling analysis of mesenchymal and non-mesenchymal tumors. Mesenchymal CRC subtype has a significant increase of innate immune cells and immune response genes related with adhesion, cell cycle, complement, leukocyte, microglial, senescence, Toll-like receptor, and transporter functions. RNA isolated from human CRC tumors was analyzed using the NanoString nCounter PanCancer immune profiling panel. **a** Profiling of tumor-associated immune-cell-type markers in mesenchymal and non-mesenchymal tumors. **b** Expression profiling of immune-related functions in each group of tumors. Expression values are expressed as log_2_. (TIF 61809 kb)

Additional file 5: Figure S5. Immunohistochemistry of eNOS in non-classified CRC tumors. eNOS is highly expressed in advanced poorly differentiated human tumors. Hyperproliferative areas showed intense staining of this isoenzyme (black arrow), whereas areas that still maintain a normal structure did not show any expression (red arrow). Scale bars: 100 μm. (TIF 16867 kb)

Additional file 6: Figure S6. Kaplan–Meier plots from an analysis of the correlation between eNOS **(a)** or iNOS **(b)** mRNA expression level and patient survival using the best separation. eNOS upregulation and low iNOS expression significantly decrease the 5-year survival in colorectal cancer, colon adenocarcinoma and rectum adenocarcinoma patients. Patients were divided into low or high groups based on the level of expression of the NOS isoform. Data were taken from The Human Protein Atlas database. (TIF 76270 kb)

Additional file 7: Figure S7. Timeline of organoid formation in control or c-PTIO (100 μM) pre-treated HCT-116 and Caco-2 tumor cells. NO scavenging with c-PTIO impairs the capacity of CRC cells to form organoids and alters the morphology of Caco-2 organoids.Scale: 100 μm. (TIF 50492 kb)

Additional file 8: Table S1. Mouse primer sequences used in this study. (TIF 45302 kb)
