# Supplementary figures and images for: A role for endothelial nitric oxide synthase in intestinal stem cell proliferation and mesenchymal colorectal cancer
Source: BMC Biol. 2018 Jan 10;16:3. doi: 10.1186/s12915-017-0472-5 (PMC5795284; doi:10.1186/s12915-017-0472-5)

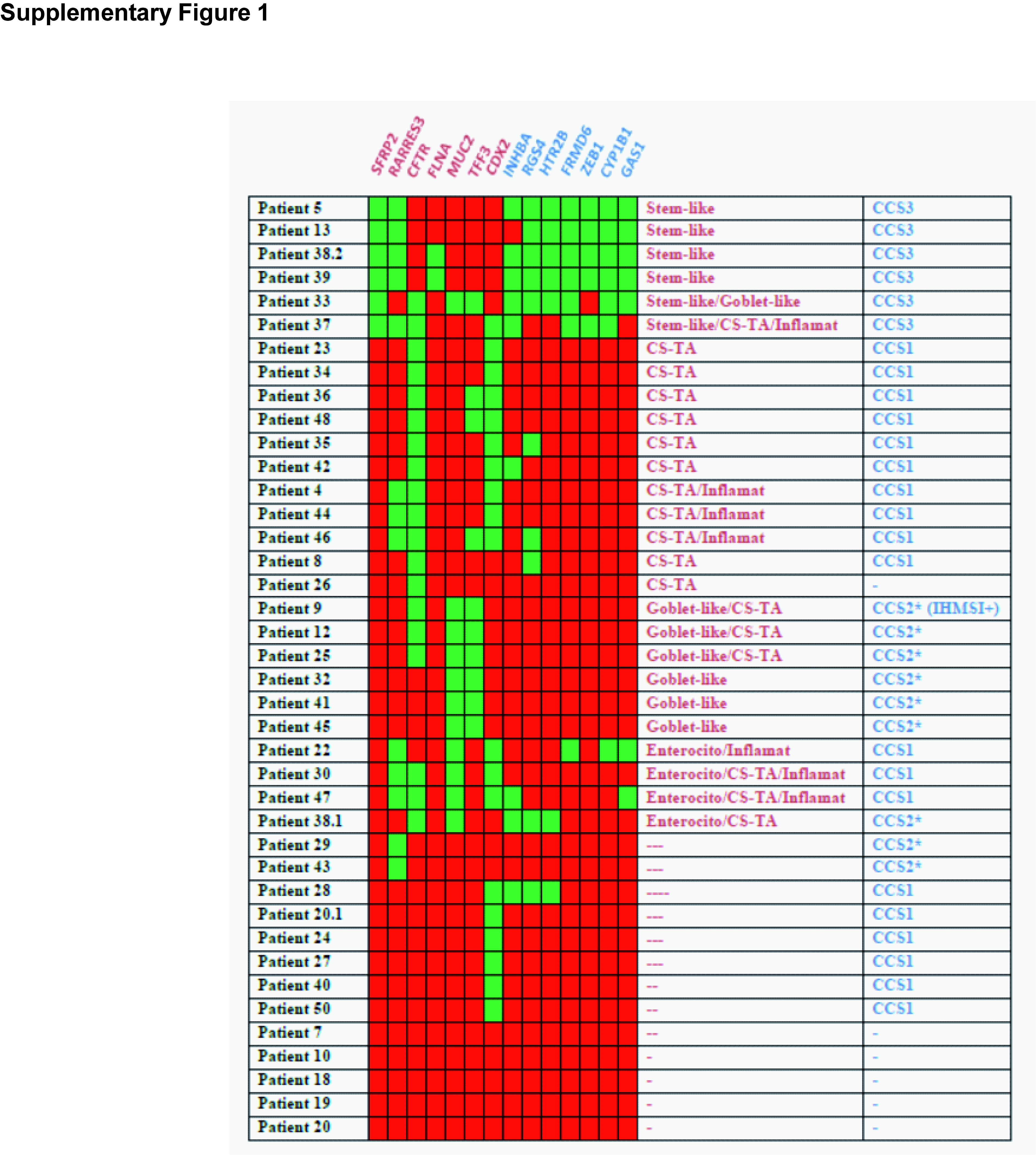

Supplement: Supplementary file 1 — Qualitative supervised classification of human CRC tumors into different CRC subtypes. Data analysis was performed using nSolver software from NanoString Technologies. Raw expression data were normalized and an overall average was established for each classifier gene, as described by Sadanandam et al. [10] and De Sousa et al. [6]. Positive expression indicates a value of expression above the average and it is shown as a green square, whereas negative expression is a value below the overall mean and it is shown as a red square. (TIF 64176 kb) [file 12915_2017_472_MOESM1_ESM.tif]

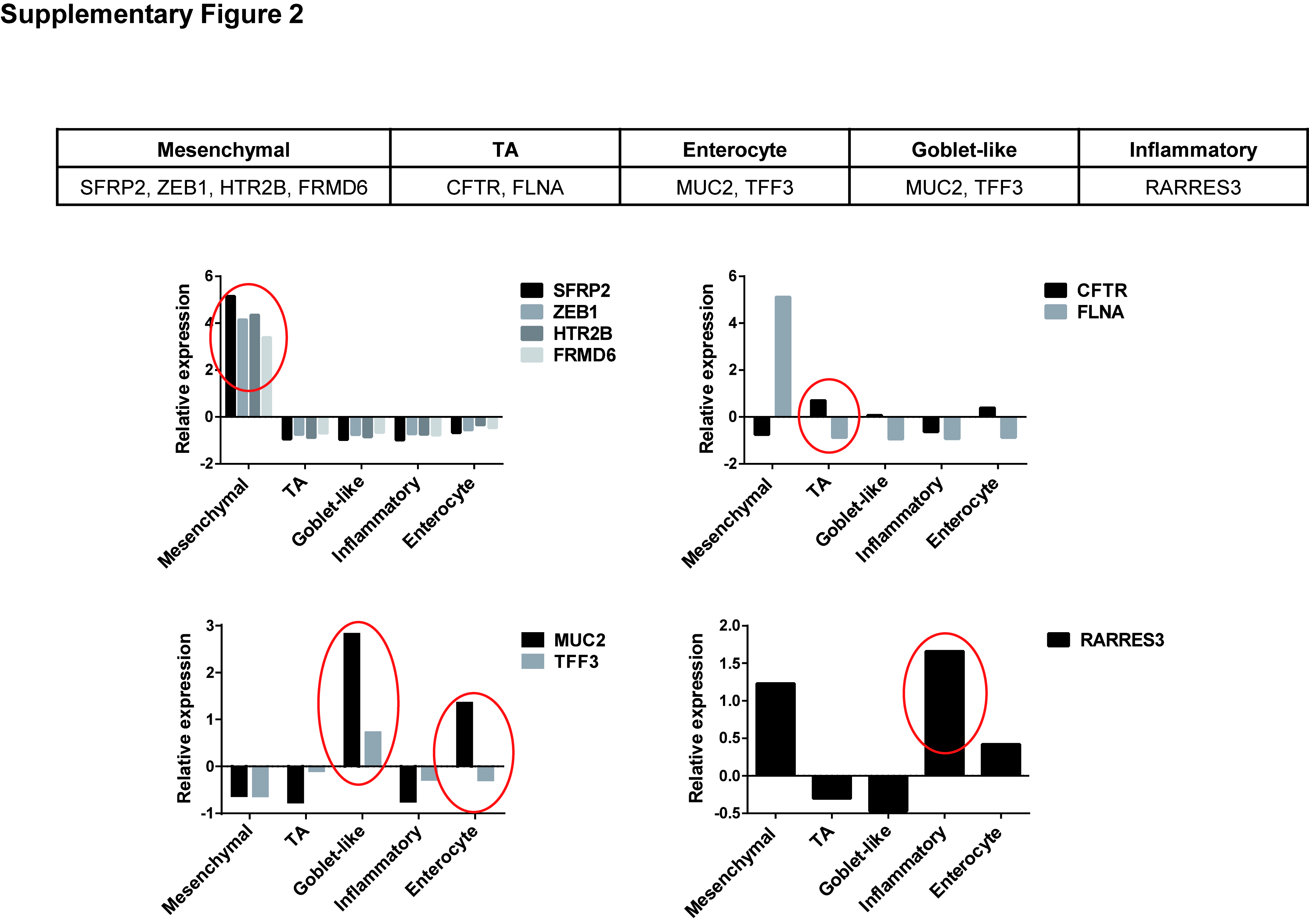

Supplement: Supplementary file 2 — Relative expression of classifier genes in all tumor samples. List of classifiers genes used and their relative expression in all tumor samples: mesenchymal, transit-amplifying, goblet-like, enterocyte, and inflammatory. Data analysis was performed as described in “Methods.” (TIF 51415 kb) [file 12915_2017_472_MOESM2_ESM.tif]

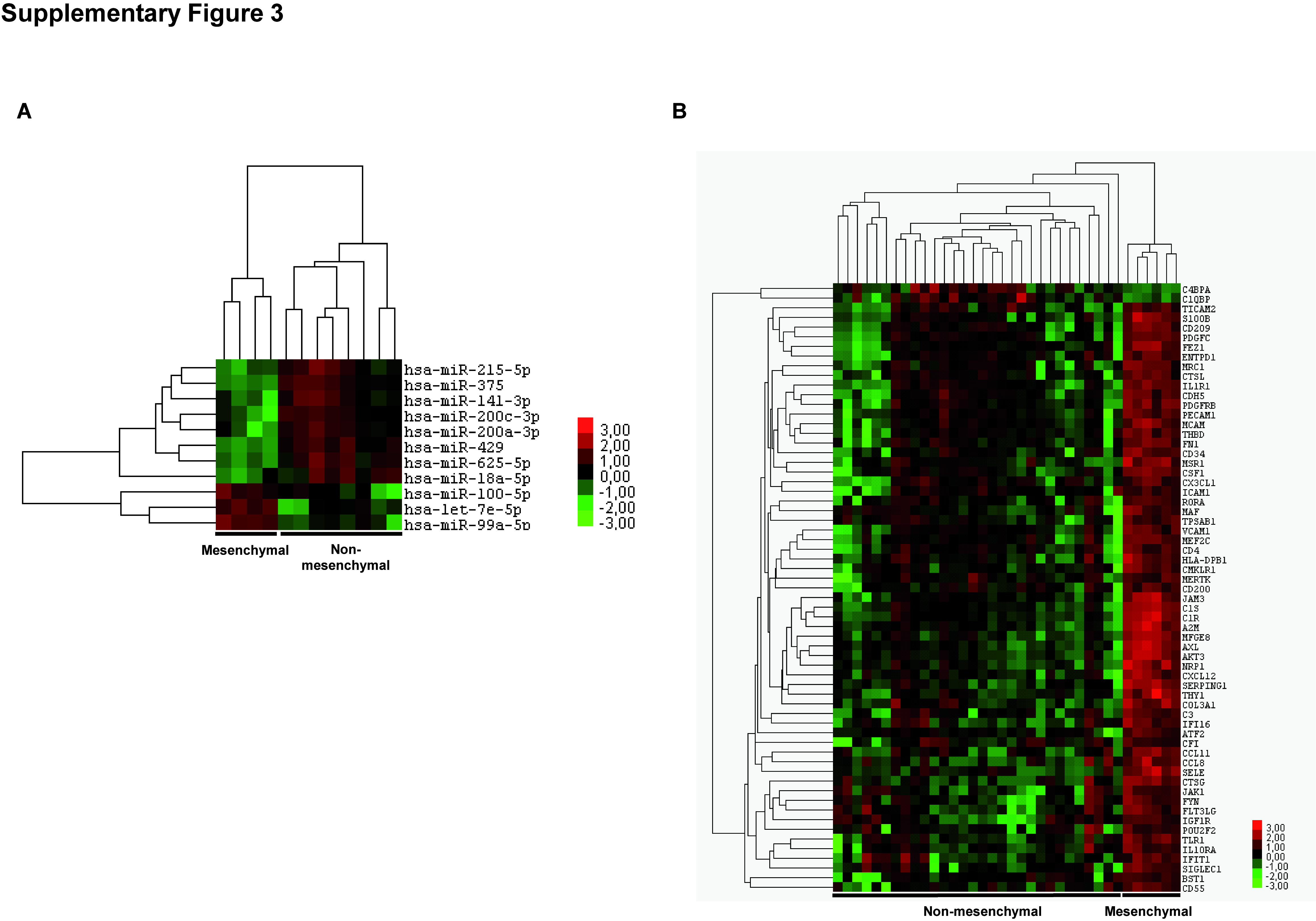

Supplement: Supplementary file 3 — The human mesenchymal CRC subtype has a specific miRNA and tumor immune response signature. Heat maps were generated using nSolver software from NanoString Technologies. a miRNA signature of mesenchymal tumors compared with other non-mesenchymal subtypes. b Tumor immune response signature of mesenchymal tumors compared with non-mesenchymal tumors. (TIF 59213 kb) [file 12915_2017_472_MOESM3_ESM.tif]

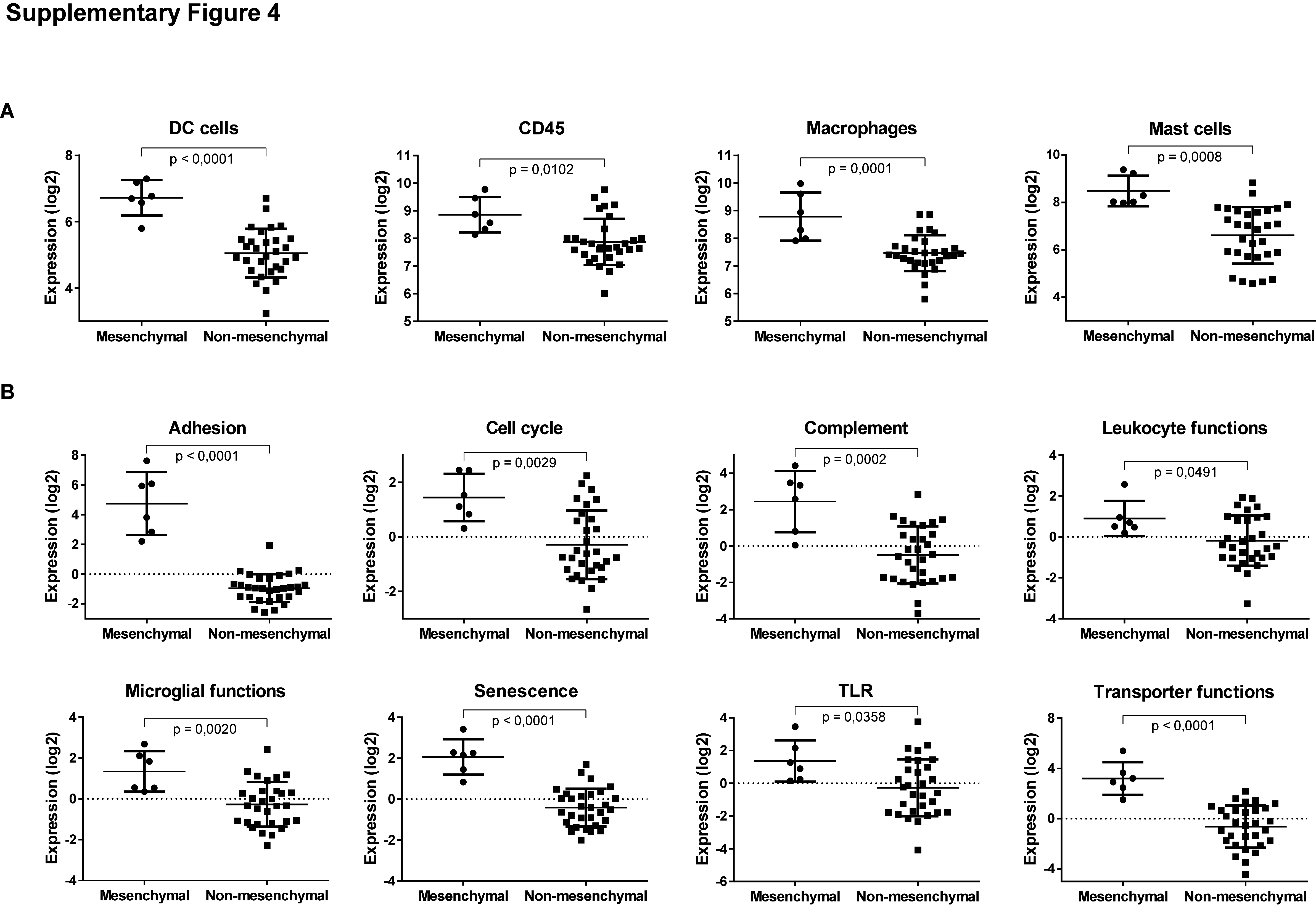

Supplement: Supplementary file 4 — NanoString immune-profiling analysis of mesenchymal and non-mesenchymal tumors. Mesenchymal CRC subtype has a significant increase of innate immune cells and immune response genes related with adhesion, cell cycle, complement, leukocyte, microglial, senescence, Toll-like receptor, and transporter functions. RNA isolated from human CRC tumors was analyzed using the NanoString nCounter PanCancer immune profiling panel. a Profiling of tumor-associated immune-cell-type markers in mesenchymal and non-mesenchymal tumors. b Expression profiling of immune-related functions in each group of tumors. Expression values are expressed as log2. (TIF 61809 kb) [file 12915_2017_472_MOESM4_ESM.tif]

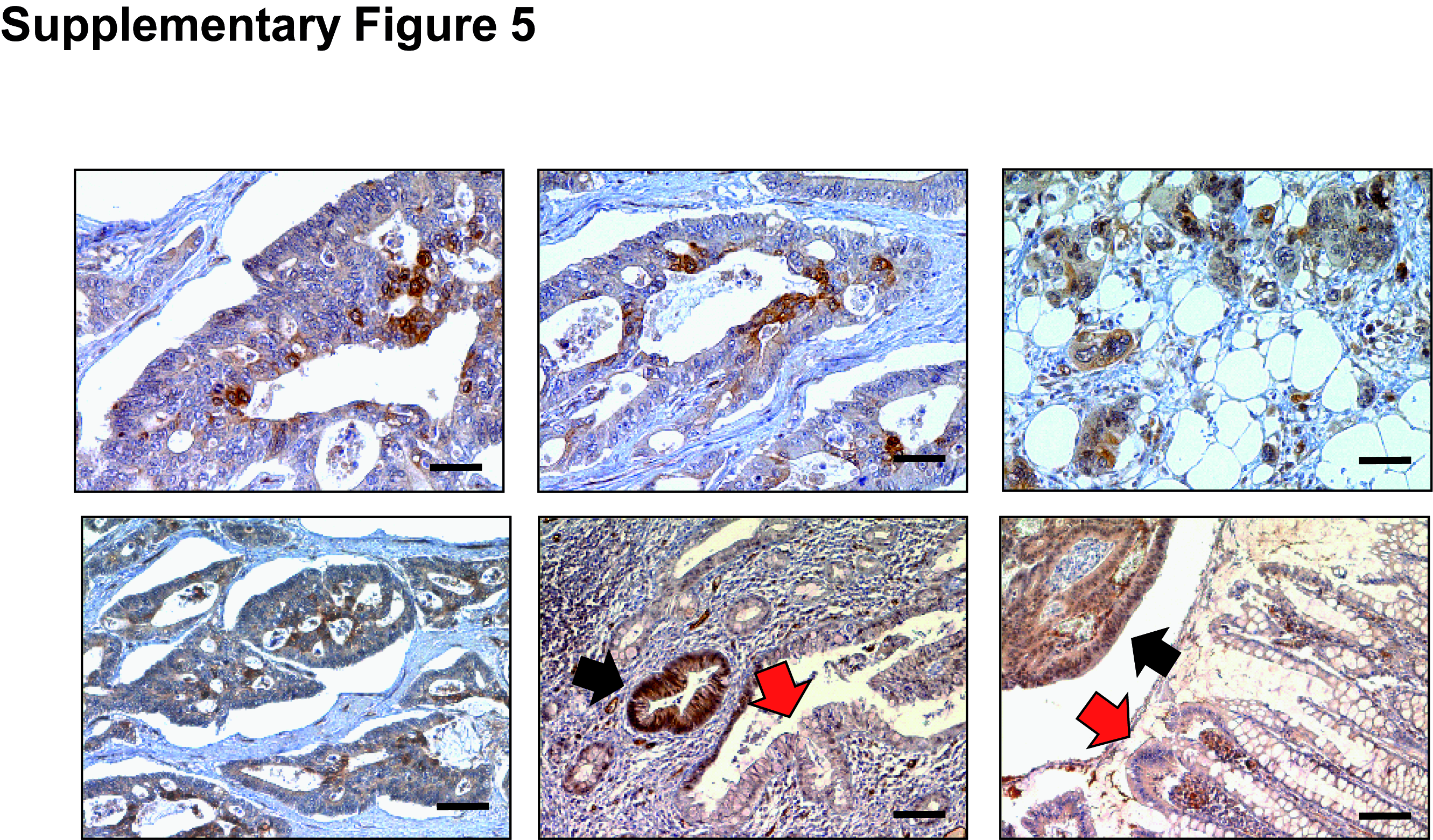

Supplement: Supplementary file 5 — Immunohistochemistry of eNOS in non-classified CRC tumors. eNOS is highly expressed in advanced poorly differentiated human tumors. Hyperproliferative areas showed intense staining of this isoenzyme (black arrow), whereas areas that still maintain a normal structure did not show any expression (red arrow). Scale bars: 100 μm. (TIF 16867 kb) [file 12915_2017_472_MOESM5_ESM.tif]

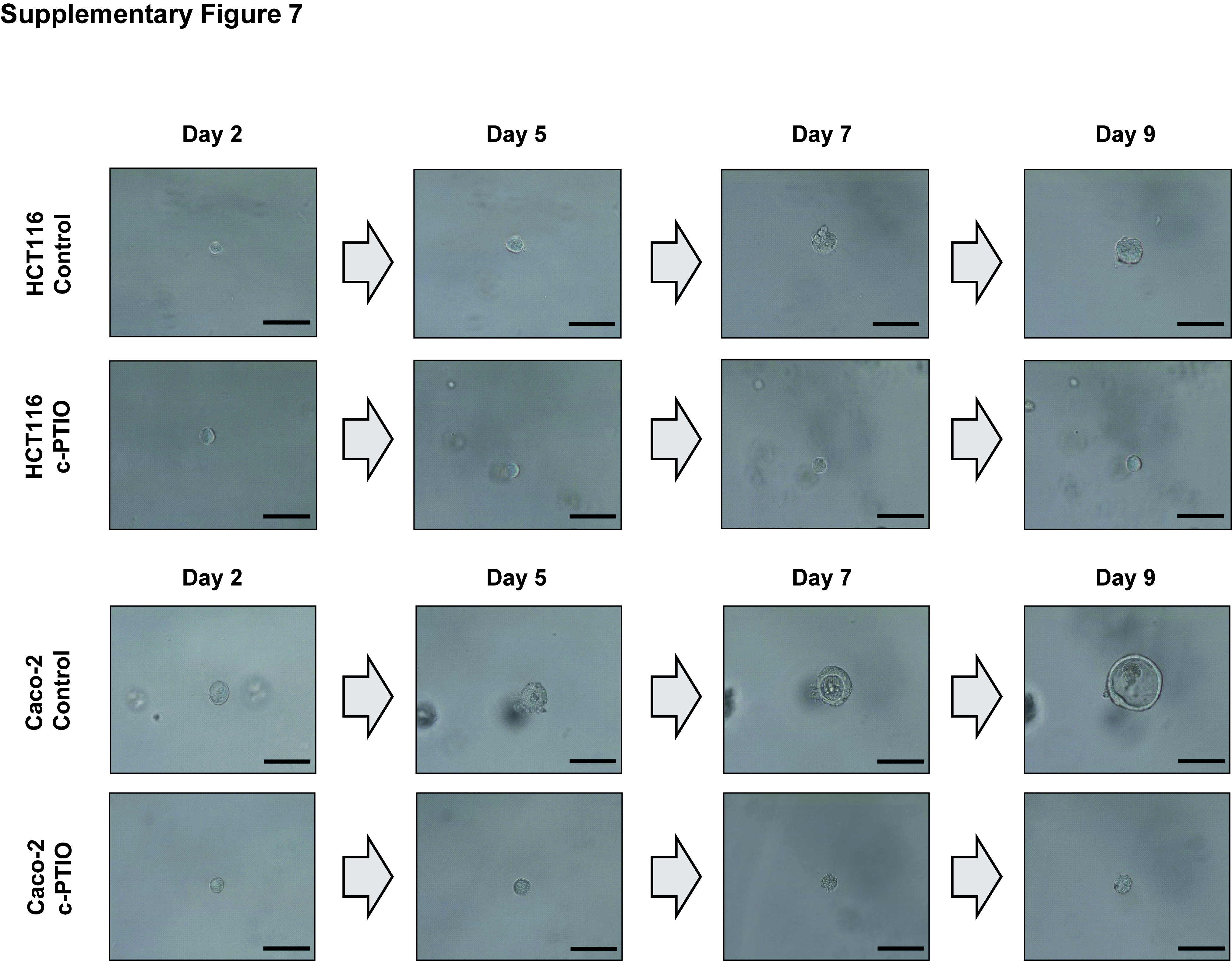

Supplement: Supplementary file 7 — Timeline of organoid formation in control or c-PTIO (100 μM) pre-treated HCT-116 and Caco-2 tumor cells. NO scavenging with c-PTIO impairs the capacity of CRC cells to form organoids and alters the morphology of Caco-2 organoids.Scale: 100 μm. (TIF 50492 kb) [file 12915_2017_472_MOESM7_ESM.tif]

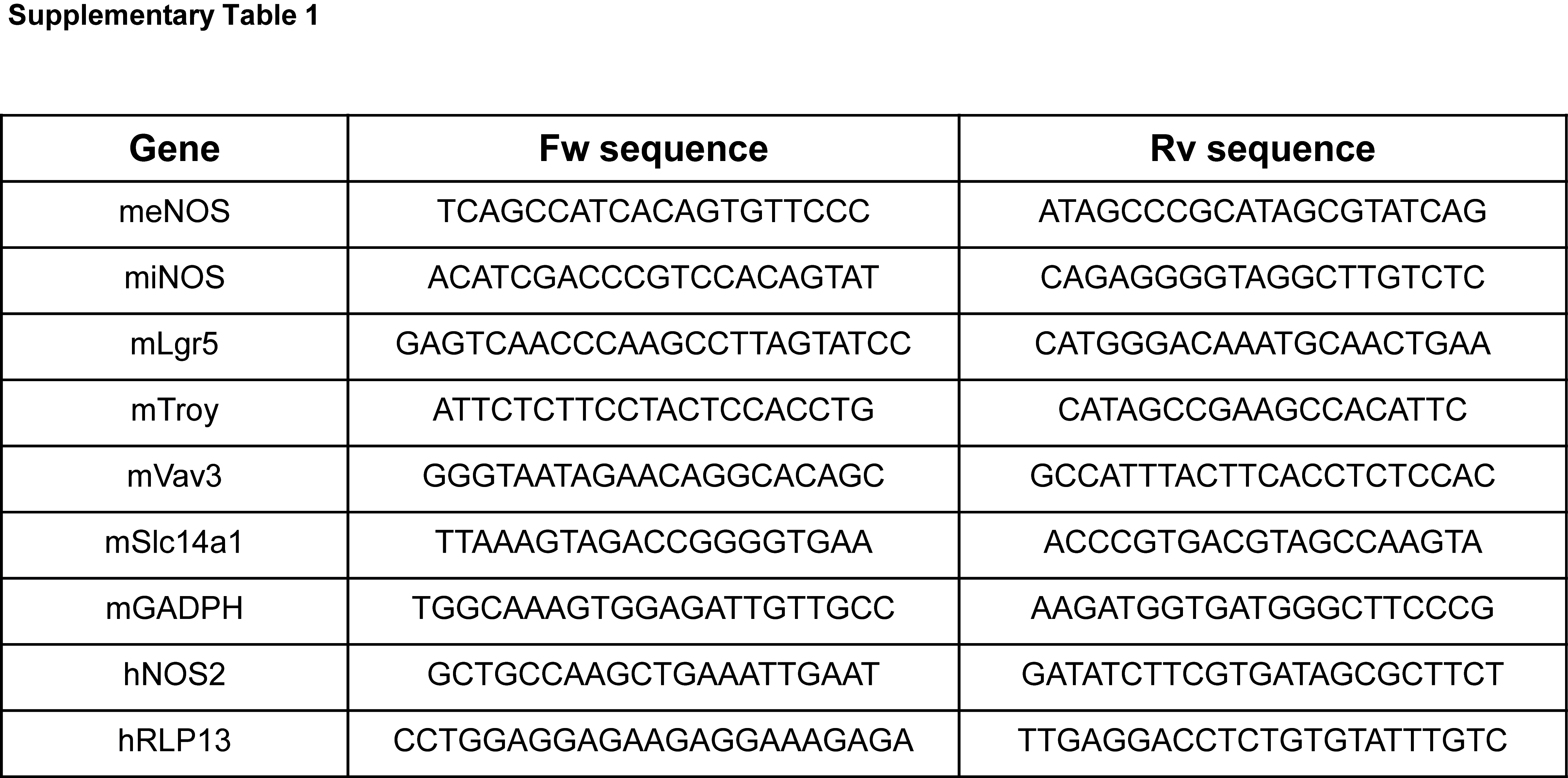

Supplement: Supplementary file 8 — Mouse primer sequences used in this study. (TIF 45302 kb) [file 12915_2017_472_MOESM8_ESM.tif]
